# Electroacupuncture at acupoint ST 37(Shangjuxu) improves function of the enteric nervous system in a novel mouse constipation model

**DOI:** 10.1186/s12906-016-1377-5

**Published:** 2016-10-18

**Authors:** Chao Liang, Kaiyue Wang, Bin Xu, Zhi Yu

**Affiliations:** 1 Nanjing University of Chinese Medicine, Nanjing, 210046 Jiangsu Province China; 2 Xi’an Traditional Chinese Medicine Brain Disease Hospital, Xi’an, 710000 China

**Keywords:** Electroacupuncture(EA), Enteric nervous system(ENS), Gastrointestinal (GI), Neuronal nitric oxide synthase (nNOS)

## Abstract

**Background:**

Electroacupuncture (EA) at acupoint ST 37 (Shangjuxu) has been used to alleviate gastrointestinal symptoms and improve gastrointestinal motility. However, the mechanisms by which EA affects the enteric nervous system (ENS) have scarcely been investigated. In this study, we investigated whether EA could improve ENS function.

**Methods:**

A constipation model was established by gastric instillation of ice-cold saline daily for 14 days. The constipated mice were divided into two groups: the model group, which was not treated, and the EA group, which received EA at ST 37 at a frequency of 2–15 HZ and an amplitude of 1 mA for 15 min a day for 3 days. A further six mice were included as a non-constipated control group. After EA treatment, intestinal propulsion and defecation time were measured. Additionally, in jejunum, ileum and proximal colon myenteric plexus, the expressions of PGP9.5 and nNOS were measured by immunohistochemistry.

**Results:**

The EA group demonstrated significant improvements in carbon propulsion rates and defecation time compared to model group (*P* < 0.05). In addition, after EA, the PGP9.5 and nNOS expression in jejunum, ileum and proximal colonic myenteric plexus was back to normal levels.

**Conclusion:**

This study suggests that EA stimulation at ST 37 is capable of ameliorating intestinal motility dysfunction, and can partly restore enteric neuron function. The ENS can participate in changes in intestinal motility by affecting inhibitory neurons.

## Background

Constipation is a symptom of underlying defects in transit of fecal mass through the gut or in defecation, and is a commonly diagnosed functional gastrointestinal (GI) disorder. Constipation is usually associated with a number of diseases, and is characterized by a series of complex GI symptoms in the absence of mechanical obstruction of the GI tract [1, 2]. Diet, side effects of medication, and hormonal disorders may induce constipation [3–5]. Therapy for constipation is largely directed towards treating the symptoms, and most of the treatment methods are uniformly effective [6]. However, these treatments do not address the underlying dysfunction in the GI tract that results in constipation.

Acupuncture as one of the most frequently applied methods in Traditional Chinese Medicine, which has a history of more than 3000 years, has gained increased popularity. In recent years, electroacupuncture (EA) at different acupoints has come to be recognized as a potential effective therapy to treat GI disorder. Preclinical researches have shown that ST 37 could increase GI transit, relieve defecation difficulty and improve life quality [7–9], and indicated that it is effective for constipation. Previous experiments have shown chronic and recurrent cold water irritation to stomach might cause long-term effects on bowel movements, which resulted in the GI motility, such as inhibited jejunal and colonic motility [10, 11]. In a cold water-induced rat model of constipation, EA stimulation at ST 37 increases faecal water content, defaecation frequency and GI transit [12, 13]. ST37 has a positive effect on objective markers of constipation. It is believed that acupuncture at different acupoints exerts different effects on internal organs to restore the homeostatic balance [14–16]. Most previous research has focused on the effects on central and peripheral neural pathways in EA’s ameliorating effects on intestinal motility [17]. However, the effects and mechanisms of EA on the enteric nervous system (ENS) have not been widely investigated. The purpose of this study was to investigate whether EA affects the ENS, and to explore local neural mechanism of EA in the gut.

## Methods

### Animals

C57BL/6 J mice (SPF-grade, 3-week-old males, 20–25 g) were purchased from the Model Animal Research Center of Nanjing University (Nanjing, China, license number: SCXK 2013–0005). Animals were housed in a room with 12 h light–dark cycle (turn on at 8:00 a.m.) maintained at 22 ± 2 °C with 60 % humidity and ad libitum access to food and water. All experimental manipulations were undertaken in accordance with the Principles of Laboratory Animal Care and the Guide for the Care and Use of Laboratory Animals, published by the National Science Council, China.

### Experimental model of constipation

The mice were randomly divided into two groups: a 0–4 °C saline-treated group (*n* = 40), and a normal feeding group (*n* = 10), randomly numbered, and raised in single cages that allowed normal access to food and water. Wire netting was used to facilitate the separation and collection of stools. The constipation model was established by gastric instillation of ice-cold (0–4 °C) saline daily for 14 days [18]. To eliminate the influence of biological rhythms, intragastric administrations were conducted at 8:00 am daily for 14 d. Animals were initially administered ice-cold (0–4 °C) saline at a dose of 0.2 mL/mouse, and then the dose was increased by 0.05 mL/mouse every 5 d. Control mice were raised normally without intragastric administration of ice-cold saline.

### Materials

Materials used in this study include wire netting (to facilitate the separation and collection of stools); a precision electronic balance (Sartorius Co, Beijing, China); ceramic cups (high temperature-resistant, radius: 2 cm, high: 3.5 cm).

Drugs included saline (Sodium chloride injection; Nanjing Chemical Reagent Co, Jiangsu, China), acacia gum (Gum Arabic powder; BASF Chemical Co, Tianjin, China), and black or red carbon powder (Color toner; Sanheng Information Technology Co). Primary antibodies for immunohistochemistry included PGP9.5 (Abcam, Cambridge, UK) and nNOS (Abcam, Cambridge, UK). The secondary antibody used was HRP-Polymer Rabbit anti-Mouse IgG (Boster Biotech, Wuhan, China).

Black and red carbon suspensions were prepared as follows: acacia gum (100 g) was added to 800 mL of water and boiled until transparent. Then the solution was mixed with black or red carbon powder (50 g) and boiled three times. After cooling, each solution was diluted with water to 1000 mL and then stored at 4 °C. The solutions were agitated prior to use.

### EA treatment

After the constipation model was successfully established, the constipated mice were randomly subdivided into two groups (*n* = 6 for each group): the model group, which remained untreated, and the EA group, which received EA at ST 37. EA stimulation was applied by two pairs of stainless steel needles (0.25 mm in diameter) inserted bilaterally at ST 37 (2 mm lateral to the anterior tubercle of the tibia and 6 mm below the knee joint) [19]. After insertion into the acupoint of constipated mice, the needles were stimulated by an EA apparatus (#HANS-100A, Nan Jing Ji Sheng Medical Treatment Science and Technology Co., China). Electrical stimulus intensity was set at 1 mA with a frequency of 2-15Hz. The stimulation was delivered for 15 min a day for 3 days.

### Measurement of intestinal function

After 12 h of fasting, mice were intragastrically administrated a suspension of black carbon (0.3 mL) and killed 10 min later via cervical dislocation. The section of intestine extending from the pylorus to the ileocecal valve was removed. The full length of the intestinal tract as well as the propulsive distance of black carbon in the tract was measured under a tension-free state, and the ratio of the propulsive distance to the length of the intestinal tract was determined for all groups. Additionally, after 12 h of fasting, another group of mice were given a red carbon suspension (0.3 mL), and the time required to defecate the first stool pellet containing the red indicator was recorded.

### Tissue preparation and immunohistochemistry

At the end of the experiment, small intestine (jejunum and ileum) and proximal colon tissue specimens were removed after 12 h of fasting. Segments approximately 1 cm in length were opened along the mesenteric border, and immediately fixed by immersion in 4 % paraformaldehyde for 24 h. Then the samples were processed for paraffin embedding in vacuum and cut at a thickness of 10 μm for immunohistochemistry.

Sections were deparaffinized in xylene and hydrated in a graded solution of ethanol. Activity of endogenous peroxidases was blocked with 3 % hydrogen peroxide. Sections were sequentially incubated in 3 % hydrogen peroxide and blocked with 5 % bovine serum albumin (BSA) for 30 min at 37 °C. The primary antibodies (Abcam, Cambridge, UK) for PGP9.5 (1:1000) and nNOS (1:800) were applied to the sections and each specimen was incubated in a moist chamber overnight at 4 °C, and then washed for three times in 0.01 mol/L phosphate-buffered saline (PBS; pH 7.2). After that, the slides were incubated with horseradish peroxidase (HRP)-Polymer Rabbit anti-Mouse IgG(Boster Biotech, Wuhan, China) for 90 min at 37 °C, then incubated in 3,3-diaminobenzidine (DAB) solution for 2-5 min. Specificity of the antibody was confirmed by negative control staining in the absence of primary antibody treatment. Two observers evaluated the slides using an Olympus FV500 optical microscope (Olympus, Tokyo, Japan). Positive immunostaining was evaluated at 5 random visual fields at a magnification of 400. The mean density of positive expression was assessed using image analysis software (Image Pro Plus 6.0).

### Statistical analysis

Data were expressed as mean ± standard error Statistical analysis was conducted using SPSS 17.0 software with one-way analysis of variance. Data were compared using the Student Newman Keuls post-hoc test. Differences where *P* < 0.05 were considered to be statistically significant.

## Results

### Effects of EA stimulation on small intestine function

**Fig. 1:**
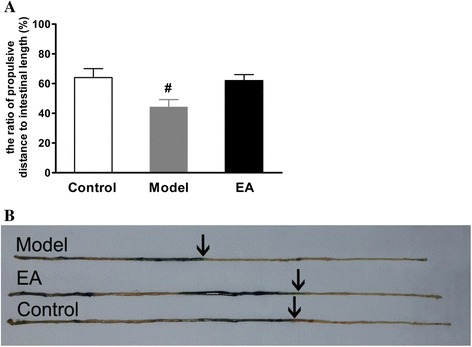
Measurement of carbon propulsion rate. **a**: All groups were used in the intestinal propulsion experiment. The distances traveled by black carbon significantly increased in the EA group compared to the Model group. **b**: Examples of intestine and black carbon trace for all three groups. (*n* = 6 for each group), ^#^ *P* < 0.05 vs. Control or EA group

Carbon intestinal propulsion experiments showed the distances by which black carbon was propelled decreased in constipation models compared to control group (Fig. 1a, *P* < 0.05). After EA treatment, carbon propulsion rates significantly increased, and did not differ from control group values (Fig. 1b).

### Effects of EA stimulation on defecation time

**Fig. 2:**
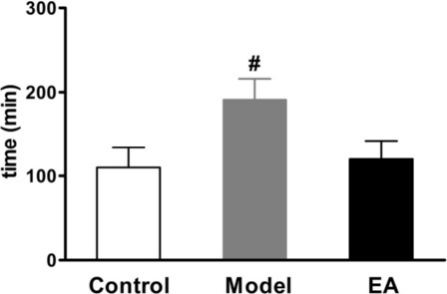
Measurement of the defecation time. All groups were used in tracking time required for defecation of the first indicator-containing stool pellet. (*n* = 6 for each group), ^#^ *P* < 0.01 vs. Control or EA group

In this experiment, the defecation time was prolonged in constipation models compared to control group, as would be expected. After EA stimulation treatment, defecation time significantly decreased compared to constipation models and almost returned to normal (Fig. 2).

### Effect of EA on total neuronal PGP9.5 protein expression

**Fig. 3:**
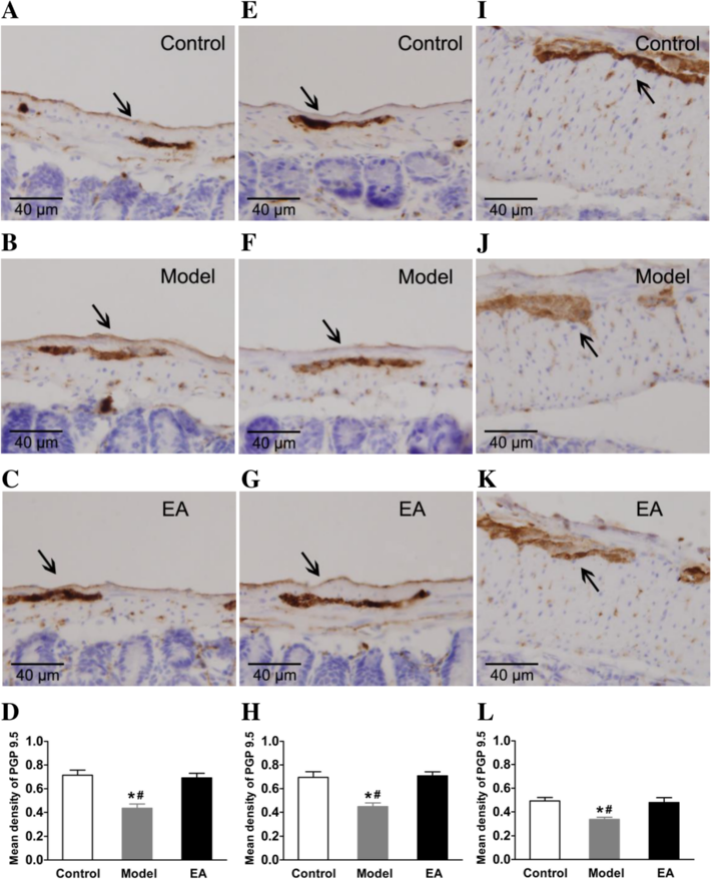
Effects on total neurons-PGP9.5 protein expression in immunohistochemical staining. Tissue with brown granular deposits indicate positive immunostaining (arrows) Scale bar: 40 μm, magnification: ×400. **a**, **b**, **c**: In jejunum; **e**, **f**, **g**: In ileum; **i**, **j**, **k**: In proximal colon; **d**, **h**, **l**: Comparison of the mean density of PGP9.5-positive expression, respectively. *P < 0.01 vs. Control group; ^#^ *P* < 0.05 vs. EA group, *n* = 5 for each group

Immunohistochemical staining revealed that the number of PGP9.5-positive cells in the constipation model was markedly decreased compared with the control group in the small intestine and proximal colon. After EA treatment, the protein expression of PGP9.5 was significantly increased compared to constipation models in jejunum (Fig. 3a, b, c), ileum (Fig. 3e, f, g) and proximal colon (Fig. 3i, j, k), but there was no difference compared with the control group, respectively (Fig. 3d, h, l).

### Effects of constipation and EA stimulation on nNOS protein expression

**Fig. 4:**
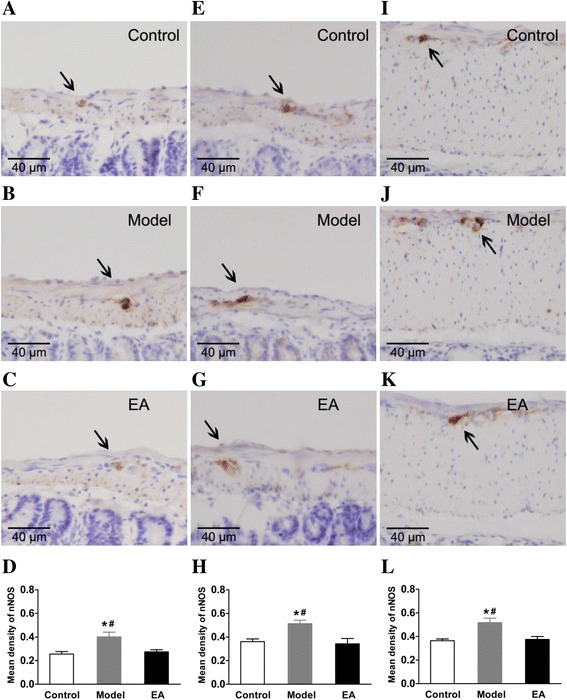
Effects on nNOS expression in immunohistochemical staining. Tissue with brown granular deposits indicate positive immunostaining (arrows) Scale bar: 40 μm, magnification: ×400). **a**, **b**, **c**: In jejunum; **e**, **f**, **g**: In ileum; **i**, **j**, **k**: In proximal colon; **d**, **h**, **l**: Comparison of the mean density of nNOS-positive expression, respectively. *P < 0.01 vs. Control group; ^#^ *P* < 0.05 vs. EA group, *n* = 5 for each group

Statistical analysis indicated that the expression of nNOS in constipation models were increased compared with the control group in the in jejunum, ileum and proximal colon. After EA at ST 37, the expression of nNOS was significantly decreased compared to constipation models in jejunum (Fig. 4a, b, c), ileum (Fig. 4e, f, g) and proximal colon (Fig. 4i, j, k), almost returned to normal levels and there was no difference compared with the control group, respectively (Fig. 4d, h, l).

## Discussion

Acupuncture is beneficial as an alternative treatment for the management of gastrointestinal functional disorders, including constipation, diarrhea and irritable bowel syndrome [20–22]. Although acupuncture has been used as an appropriate adjunct treatment for GI dysfunction disorders, the underlying mechanisms of EA on the ENS have not been well studied.

In this study, the results indicate that EA at ST 37 improved impaired intestine functions, accelerating GI motility. Acupuncture is recognized to have regionally specific effects [23]. ST 37 is located in the hind limb, and some studies have demonstrated that acupuncture at the hind limb increases GI motility [24]. In clinical treatment, ST 37 can improve GI motility and alleviate symptoms, which is effective for treating constipation [25, 26]. Previous research has focused on the effects of acupuncture on central and peripheral neural pathways; EA stimulation of points on the abdomen can influence sympathetic nerves to the GI tract, while points in the four limbs can influence parasympathetic nerves [27, 28]. Parasympathetic nerves can promote GI peristalsis, while sympathetic nerves inhibit GI movement [29]. Accordingly, the effect of stimulation at ST 37 support these previous results. Stimulation at ST 37 may increase the parasympathetic-sympathetic balance and promote GI movement. As the ENS plays an important role in the GI motility, we hypothesized that the ENS could participate in EA stimulation of GI function. The ENS is a complex network of neurons and glia that resides in the myenteric and submucosal plexus of the bowel, and controls many aspects of bowel function [30]. The myenteric plexus, located between longitudinal and circular muscle, primarily controls muscle contraction and relaxation [31]. Therefore, ENS defects may underlie common GI motility problems such as constipation.

Our previous study indicated that irritation with ice-cold saline caused changes in the ENS in the jejunum, ileum and proximal colonic myenteric plexus [18]. Indeed, immunohistochemistry results showed the protein expression of PGP9.5 after EA stimulation was significantly increased compared to constipation models in the jejunum, ileum and proximal colon myenteric plexus. PGP9.5 is a neuron-specific protein, which can be used to accurately locate enteric neurons and indicate ENS function [32]. In this study, our results indicated that EA may improve enteric neurons and repair the impaired ENS. A previous study found that EA at ST 36 (Zusanli), which is also located in the hind limb, can induce regeneration of lost enteric neurons in a diabetic model [33]. Thus, the enteric neurons in the ENS are likely to be affected by EA stimulation. As we know, abnormal activity of the ENS can have significant effects on the functions of the digestive tract, and many diseases illustrate essential roles of the ENS [34, 35]. In our opinion, EA ameliorates intestinal motility impairment through both central and peripheral neural pathways, which works in concert with the ENS.

Furthermore, the expression of neuronal nitric oxide synthase (nNOS) in the constipation model was significantly increased compared with the control group, and returned to almost normal after EA treatment. In the ENS, nitric oxide (NO) is a major inhibitory neurotransmitter, and is synthesized by neuronal NOS [29]. It has been suggested that, in the ENS, structural abnormalities of the myenteric and submucosal plexus and an abnormal neurotransmitter content have been considered to be responsible for primary chronic constipation [36]. Studies on different parts of the intestine reveal the participation of NO in the regulation of spontaneous contractions, such as colonic contractile activity [37, 38]. We concluded from our experiments that increased NO in the myenteric plexus might reduce intestinal motility, and be closely related to the development of constipation. In our view, EA at ST 37 can restore the balance of inhibitory neurotransmitters of the enteric neurons, leading to improvement of the impaired ENS function.

## Conclusion

In summary, EA stimulation at ST 37 is capable of ameliorating intestinal motility dysfunction in a mouse constipation model, and can partly restor e enteric neuron function. The ENS can participate in changes in intestinal motility by affecting inhibitory neurons. EA could effects on internal organs, and restore the homeostatic balance via central and peripheral neural pathways working in concert with the ENS.

## Abbreviations

EA: Electroacupuncture
ENS: Enteric nervous system
GI: Gastrointestinal
nNOS: Neuronal nitric oxide synthase
NO: Nitric oxide
